# Stroke risk prediction by color Doppler ultrasound of carotid artery-based deep learning using Inception V3 and VGG-16

**DOI:** 10.3389/fneur.2023.1111906

**Published:** 2023-02-14

**Authors:** Shan-Shan Su, Li-Ya Li, Yi Wang, Yuan-Zhe Li

**Affiliations:** ^1^Department of Ultrasound, The Second Affiliated Hospital of Fujian Medical University, Quanzhou, China; ^2^Department of Computed Tomography and Magnetic Resonance Imaging (CT/MRI), The Second Affiliated Hospital of Fujian Medical University, Quanzhou, China

**Keywords:** color Doppler ultrasound images, stroke risk prediction, deep learning, transfer learning, carotid artery

## Abstract

**Purpose:**

This study aims to automatically classify color Doppler images into two categories for stroke risk prediction based on the carotid plaque. The first category is high-risk carotid vulnerable plaque, and the second is stable carotid plaque.

**Method:**

In this research study, we used a deep learning framework based on transfer learning to classify color Doppler images into two categories: one is high-risk carotid vulnerable plaque, and the other is stable carotid plaque. The data were collected from the Second Affiliated Hospital of Fujian Medical University, including stable and vulnerable cases. A total of 87 patients with risk factors for atherosclerosis in our hospital were selected. We used 230 color Doppler ultrasound images for each category and further divided those into the training set and test set in a ratio of 70 and 30%, respectively. We have implemented Inception V3 and VGG-16 pre-trained models for this classification task.

**Results:**

Using the proposed framework, we implemented two transfer deep learning models: Inception V3 and VGG-16. We achieved the highest accuracy of 93.81% by using fine-tuned and adjusted hyperparameters according to our classification problem.

**Conclusion:**

In this research, we classified color Doppler ultrasound images into high-risk carotid vulnerable and stable carotid plaques. We fine-tuned pre-trained deep learning models to classify color Doppler ultrasound images according to our dataset. Our suggested framework helps prevent incorrect diagnoses caused by low image quality and individual experience, among other factors.

## 1. Introduction

With the advent of aging, cerebrovascular disease has become one of the world's three significant causes of death and disability (1). Atherosclerosis is a systemic and progressive disease, and its progression is reflected in the transformation of stable and unstable lipid plaques in the arterial lumen. Cerebrovascular disease is closely related to carotid atherosclerotic plaque. The vulnerability of carotid plaque is a significant risk for the recurrence of cerebral infarction (2). The recurrence rate of ischemic cerebral infarction patients within 7 days is as high as 8.1% (3). Carotid color Doppler ultrasound is a routine method for examining carotid plaque. Conventional two-dimensional ultrasound can accurately determine the plaque's location, size, shape, and echo and observe the degree of carotid artery stenosis and even the ulcer on the plaque surface and intraplaque hemorrhage (4). Different echoes represent changes in plaque composition, and plaque stability can be preliminarily judged according to plaque morphology, echoes, thickness, and integrity of the fibrous cap. Studies have shown that hypoechoic plaques are more prone to stroke than iso-echoic and hyperechoic plaques (5). However, the detection of ulcer plaque and intraplaque hemorrhage by conventional ultrasound is limited, and the nature of plaque mainly depends on the operator's experience and subjective judgment, which has certain limitations (6). Multiple studies used numerical simulations to analyze the fluid–structure interaction between the blood vessel wall and blood flowing through elastic arteries with eccentric stenotic plaque (7). With the rapid development of inspection techniques, contrast-enhanced ultrasound and ultra-microvascular imaging techniques are able to display new blood vessels with plaques, better assess the vulnerability of plaques, and improve the predictive value of cerebral infarction recurrence (8, 9). However, due to the limitations of inspection instruments and contrast media, contraindications cannot be used as routine screening.

Numerous imaging approaches, such as 3D imaging, auto-fluorescence imaging (AFI), and narrow-band imaging (NBI), have been developed to enhance the diagnostic system and get over the constraints listed above (10). A precise 3D reconstruction and modeling (11) of the segmented cardiac structures (12) is crucial because hemodynamic modeling of these structures aids in the evaluation of blood dynamics. There is still a need for a computer-aided autonomous framework to improve the efficiency and quality of diagnosis in daily clinical practice (13). Deep learning technology has recently permeated several areas of medical study and has taken a center stage in modern science and technology.

Deep learning technology can fully utilize vast amounts of data, automatically learn the features in the data, accurately and rapidly support clinicians in diagnosis, and increase medical efficiency. Traditional machine learning and deep learning methods in medical image analysis have been widely used in medical image diagnosis (14), and ensemble learning techniques are also used in various medical examinations (15). For the automatic segmentation of images in cardiac radiography, Song et al. (16) utilized the deep learning technique and obtained significant results. To segment the carotid plaque in ultrasound longitudinal B-mode images, Meshram et al. (17) used U-Net architecture, where the dilated convolution layers were used in the bottleneck. Savaş et al. (18) used a multi-hidden layer neural network to detect and classify intima-media thickness and achieved 89.1% accuracy. In their experiment, they used U-Net architecture to segment the same plaque manually, and the results showed the Dice coefficients of 0.55 for automatic segmentation and 0.84 for semi-automatic segmentation. Pre-trained deep learning models based on massive datasets have demonstrated their superiority to conventional approaches as the processing capacity of modern hardware continues to grow. Therefore, from a deep learning perspective, transfer learning can be used to solve the image categorization problem. The study found that the transfer learning and convolutional neural network technique achieve several cutting-edge achievements in medical image analysis (19). Chatterjee et al. (20) used MobileNet and various feature selection techniques to determine the amount of plaque in the carotid artery to predict the heart risk and achieved 95% accuracy on the validation set.

We utilized the benefits of pre-trained deep learning models to enhance the diagnosis and overcome the mentioned limitations. Our deep learning framework improves the carotid color Doppler ultrasound for examining carotid plaque using MRI scans. We used pre-trained models, including VGG-16, ResNet-50, and Inception V3, and adjusted their hyperparameters to fit our classification task.

The study is organized as follows: Section 2 of this study illustrates the methodology, including the dataset description, feature extraction, and implementation detail of deep learning models. Section 3 explains the results and discussion. Section 4 presents the conclusion and possibilities for future research.

## 2. Materials and methods

**Figure 1:**
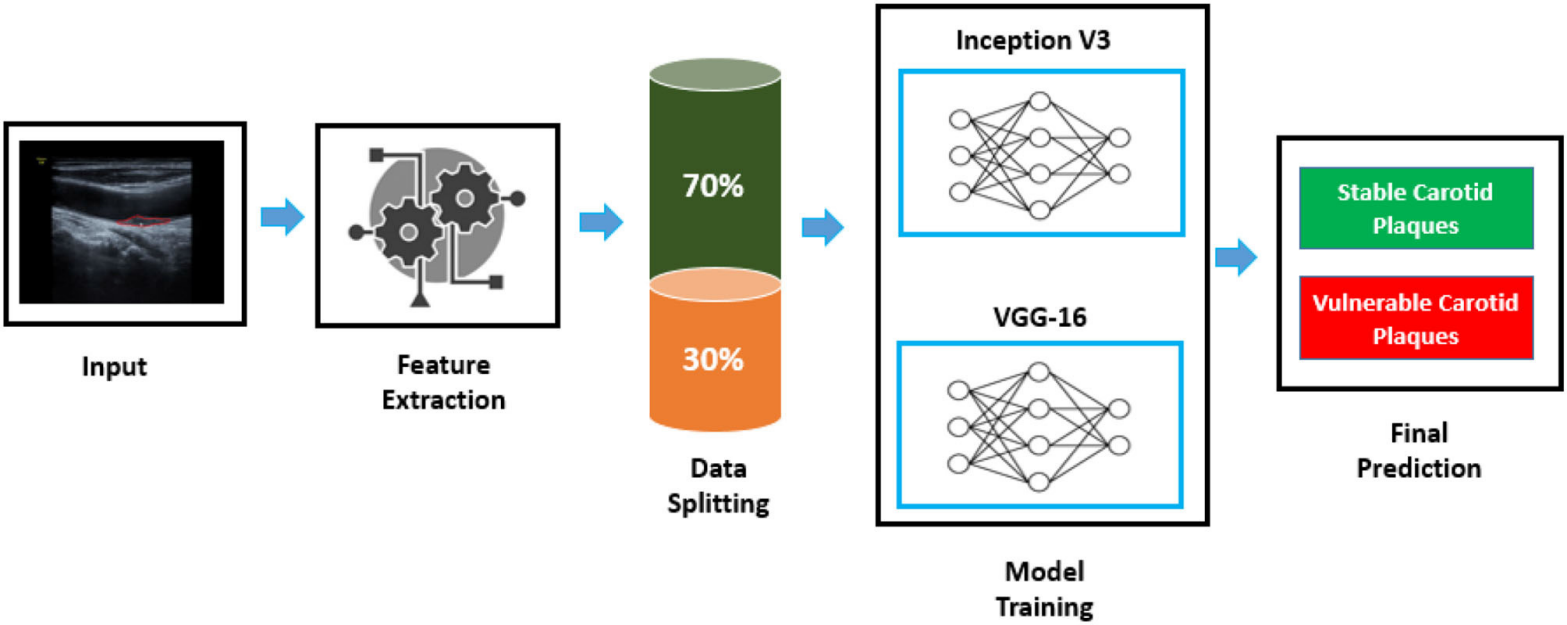
Proposed framework to classify color Doppler ultrasound images. An image is fed to the feature extraction module of a neural network; then, data are split to the train and test set. Inception V3 and VGG-16 are trained according to the dataset to classify images into two classes.

A computer-aided autonomous framework is needed to classify color Doppler ultrasound images into two types to enhance carotid plaque diagnosis. Deep learning technology has recently permeated several areas of medical study and has taken a center stage in modern science and technology (21). Deep learning technology can fully utilize vast amounts of data, automatically learn the features in the data, accurately and rapidly support clinicians in diagnosis, and increase medical efficiency. Our research implemented a deep learning framework based on transfer learning to classify color Doppler ultrasound images into vulnerable and stable carotid plaques. We used VGG-16 and Inception V3 pre-trained models, fine-tuned them, and adjusted hyperparameters according to our classification problem. The proposed framework to address the mentioned research gap is shown in Figure 1.

### 2.1. Data collection and statistics

**Figure 2:**
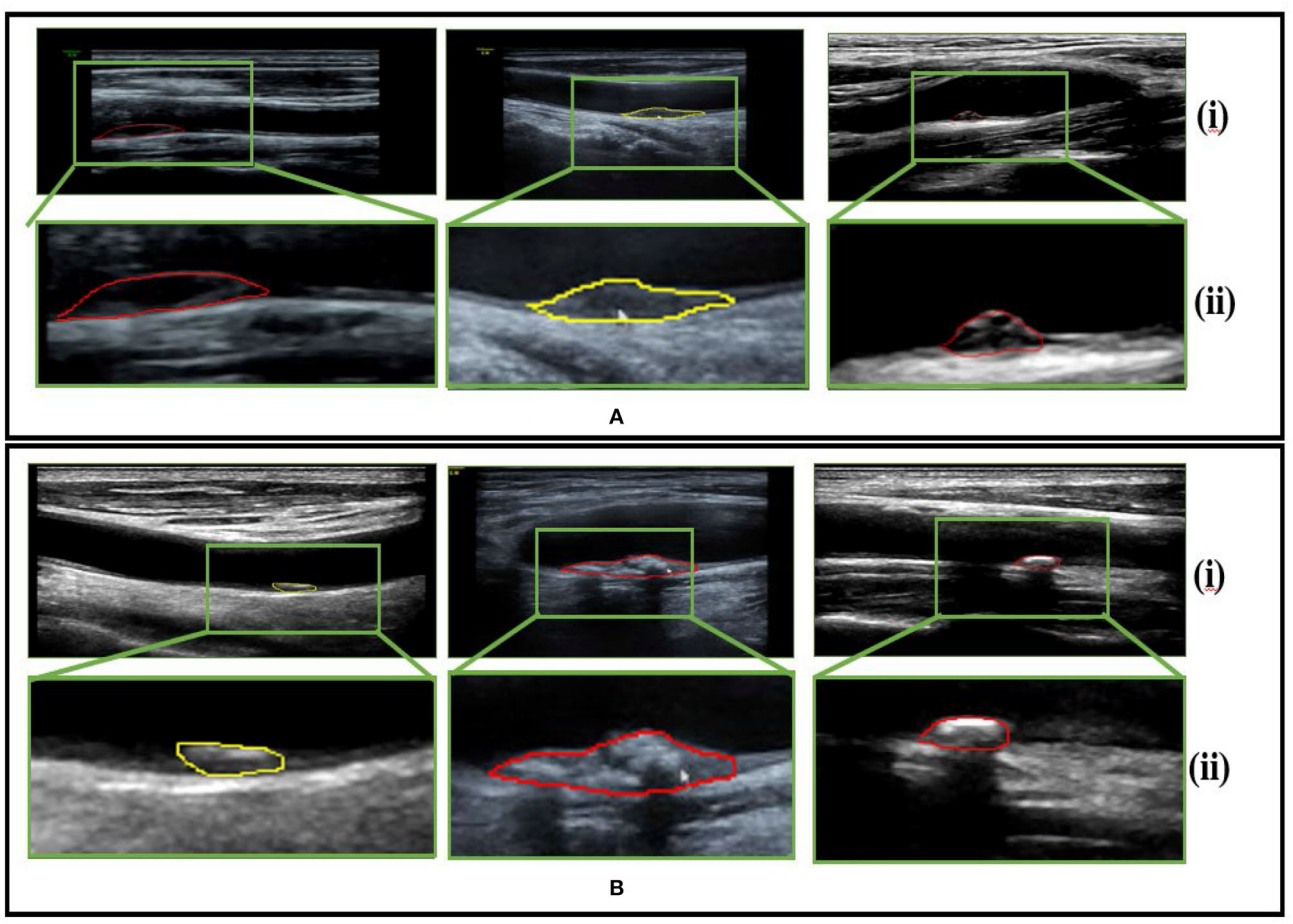
Sample dataset **(A)** pertains to vulnerable carotid plaque ultrasounds images scans and **(B)** belongs to stable carotid plaque ultrasounds images scans, whereby (i) and (ii) represent the color Doppler image scans and their respective enlarged views for clarity.

The data were collected from the Second Affiliated Hospital of Fujian Medical University, including stable cases and vulnerable cases. A total of 87 patients with risk factors for atherosclerosis in the mentioned hospital were selected. Due to the complexity of medical images and the requirement for extremely high accuracy of results, the current analysis of medical images is mainly performed by experienced personnel. We randomly selected 230 sample images of vulnerable carotid plaque from color Doppler ultrasound images, and similarly, 230 images were selected randomly for the stable carotid plaque category. We divided the dataset in the ratio of 70 and 30% for training and testing, respectively. The sample data are shown in Figure 2.

Furthermore, the dataset statistics are shown in Table 1 for a better understanding.

**Table 1:** Statistics of our dataset in each category.

**Category**	**Total number of images**
Vulnerable carotid plaque	230
Stable carotid plaque	230

The carotid artery color ultrasound confirmed the presence of the carotid artery homogeneous hypoechoic plaque (with the sternocleidomastoid muscle as the reference, the echo was slightly lower than sternocleidomastoid echoes and may have hyperechoic fibrous caps) or heterogeneous hypoechoic plaque (the echoes are marginally lower than those of the sternocleidomastoid muscles, mainly hypoechoic, with hyperechoic and is echoic parts <25 %). Patients with diabetes, hypertension, and hyperlipidemia should receive regular symptomatic treatment.

Two-dimensional ultrasound diagnostic criteria for vulnerable plaques are as follows: The overall shape of the plaque is irregular, the fibrous surface cap is thin or not smooth, and the internal lipid core is low or low to anechoic, heterogeneity, and basal lines. The echo-like continuity is poor or inconsistent, or there is an ulcer on the surface of the plaque. The RESONA 7OB diagnostic ultrasound system (Mindray Medical International, Shenzhen, Guangdong Province, China) linear array probe is in the frequency of 3–11 MHz, and the patient is in a supine position with the occiput removed, with the head slightly turned to one side (to avoid hyperextension), and conventional color Doppler ultrasound of the carotid artery is performed, the thickness of plaque was observed according to the characteristics of the first echo. Homogeneous or heterogeneous hypoechoic plaques were selected as representative plaques. If the plaques were multiple, the homogeneous plaque with the lowest visual echo was selected. After determining the target plaque, the long-axis section of the plaque is taken; then, the probe is kept steady, and the image is acquired.

### 2.2. Feature extraction

We begin with a pre-trained model for extracting characteristics and changing the weights of the bottom layer, from which we obtain predictions. It is called feature extraction because we change the output layer and apply the pre-trained CNN as a fixed feature extractor (22). As the number of convolution steps increases, convolution neural networks successfully learn the edge features of the input image and some or all objects—high-level semantic information. In the convolution neural network, the convolution layer and complete connection layer can be used to extract the image's deep features; however, the convolution layer contains several dimensions, making it difficult to calculate the dimensionality reduction that comes next (23). The last layer consists of the softmax layer that computes the categorical cross-entropy loss function (*E*) between the symptomatic and asymptomatic classes and is mathematical, as given in Equation 1.


(1) $$\begin{array}{l} E=\;-\left(y^{*}\log\left(p\right)\right)+\left[{\left(1-y\right)}^{*}\log\left(1-p\right)\right] \end{array}$$

where *y* is the binary indicator for the observed class, “^*^” denotes the product, and *p* is the predicted probability of the plaque belonging to a specific class computed using deep learning models.

### 2.3. Inception V3

**Figure 3:**
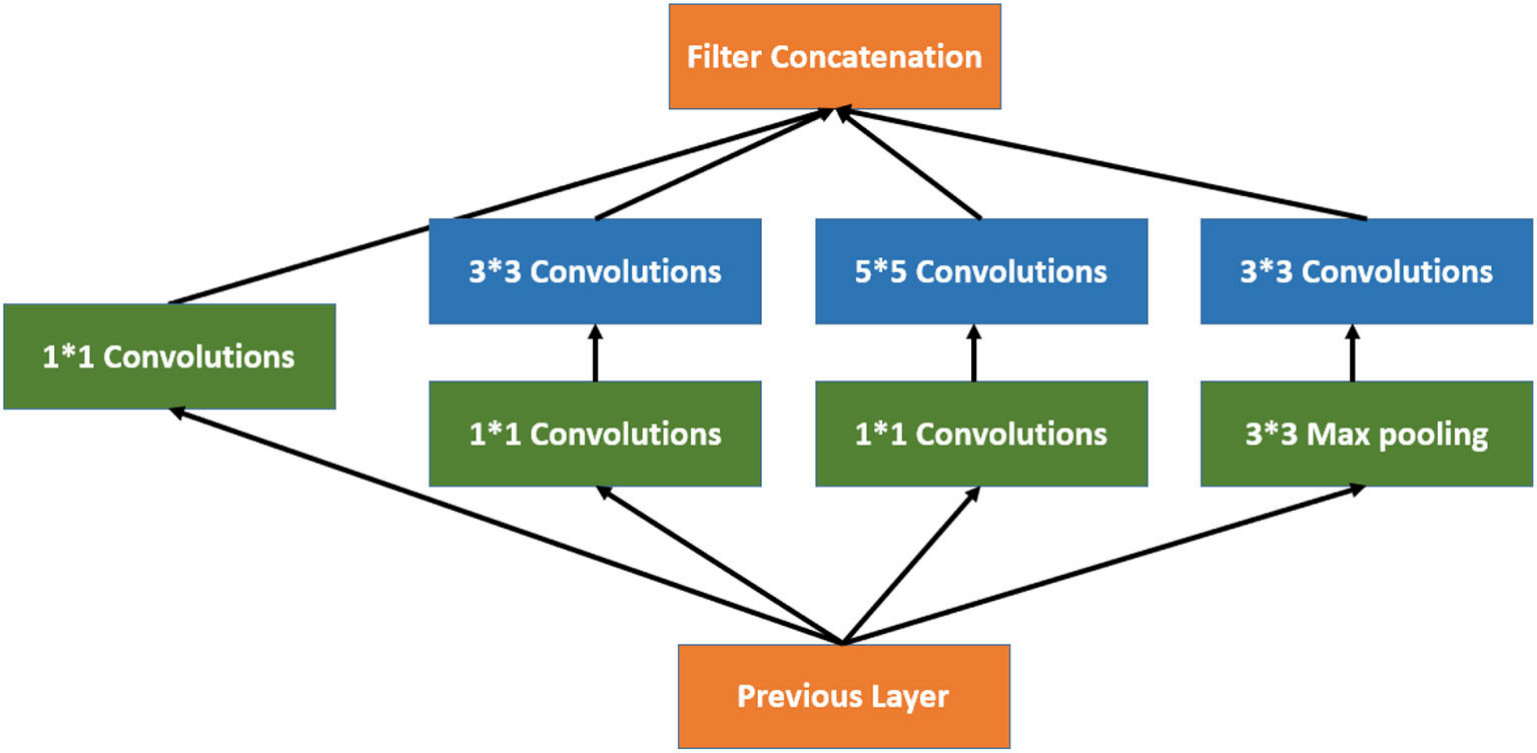
Architecture of inception network.

Inception V3 is a 48-layer deep pre-trained convolutional neural network model, as shown in Equation 2. This network was trained using a subset of the more than a million images in the ImageNet collection. Typically, the inception module includes one maximum pooling and three convolutions of various sizes (24). After the convolution operation, the channel is aggregated for the network output of the preceding layer, and the non-linear fusion is then carried out. In this model, overfitting can be avoided while enhancing the network's expression and flexibility to various scales, as shown in Figure 3.


(2) $$\begin{array}{l} A_{X}=\;{\left[\begin{array}{ccc} A_{1,1} & \ldots & A_{1N}\\ A_{21} & \ldots & A_{2N}\\ A_{M1} & \ldots & A_{MN} \end{array}\right]}^{*}\left[\begin{array}{ccc} B_{1,1} & \ldots & B_{1N}\\ B_{21} & \ldots & B_{2N}\\ B_{M1} & \ldots & B_{MN} \end{array}\right]\;\\ \;=\;\sum_{i=0}^{M-1}\;\sum_{j=0}^{N-1}A_{\left(M-1\right),\left(N-j\right)}B_{\left(i+1\right),\;\left(j+1\right)} \end{array}$$

By flattening the output layer, reducing its dimensions to one, and adding a sigmoid layer for classification along with a fully connected layer with 1,024 hidden units, with a ReLU activation function, as shown in Equation 3, and a dropout rate of 0.4, we were able to use Inception V3 by avoiding its overfitting. The neuronal weights of the classification layers are initialized with the algorithm described in (25), as shown in Equation 4.


(3) $$\begin{array}{lll} f\left(x\right) & = & \max\left(0,\;x\right) \end{array}$$

(4) $$\begin{array}{lll} W_{k}\; & \sim & \;U\left(-\frac{1}{\sqrt{m}},\frac{1}{\sqrt{m}}\right) \end{array}$$

where e *U* (–*a, b*) is a uniform distribution in the interval [–*a, b*], m is the size of the previous layer, and *W*_*k*_ stands for weight parameters in the CNN at iteration *k*. The complete model architecture and hyperparameter details are shown in Table 2.

**Table 2:** Hyperparameter details used in the Inception V3 model according to our dataset.

**Layer (type)**	**Output shape**	**Param**
inception_v3 (Model)	(None, 8, 8, 2,048)	21,802,784
flatten_ 1 (Flatten)	(None, 131,072)	0
activation_95 (Activation)	(None, 131,072)	0
dropout_ 1 (Dropout)	(None, 131,072)	0
dense_ 1 (Dense)	(None, 1,024)	134,218,752
activation_96 (Activation)	(None, 1,024)	0
dropout_2 (Dropout)	(None, 1,024)	0
dense_2 (Dense)	(None, 28)	28,700
activation_97 (Activation)	(None, 2)	0

### 2.4. VGG-16

**Figure 4:**
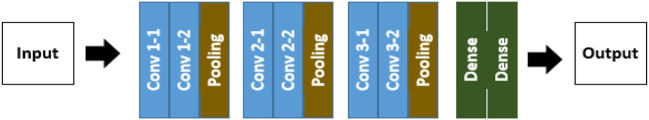
Network architecture of VGG-16.

The convolution neural network model known as the VGG-16 neural network was created and trained by the Visual Geometry Group (VGG) at the University of Oxford (26). The architecture structure of VGG-16 is shown in Figure 4, which consists of convolutions, pooling, and dense layers. A layer where *y* = *f (x)* should be considered. We are interested in learning which *x* components affect which *y* components.

In addition, we consider this to be the receptive field. As a result, the output component *y*_*i*_, *j* is solely dependent on the input components *x*_*i*_, *j*, where (i, j) ∈ Ω (*i*″, *j*″). The set Ω (*i*″, *j*″) rectangle is defined by Equations 5 and 6.


(5) $$\begin{array}{l} i\;\in \;\alpha \left(i^{\prime \prime }\;-\;1\right)\;+\;\beta_{h}+\;\left[-\;\frac{\delta_{h}-1}{2},\;\frac{\delta_{h}-1}{2}\right] \end{array}$$

(6) $$\begin{array}{l} i\;\in \;\alpha \left(j^{\prime \prime }\;-\;1\right)\;+\;\beta_{v}+\;\left[-\;\frac{\delta_{v}-1}{2},\;\frac{\delta_{v}-1}{2}\right] \end{array}$$

where (α_*h*_, α_*v*_) is the stride, and (β_*h*_, β_*v*_) and (Δ_*h*_, Δ_*v*_) are the offset and respective field size, respectively. We enhanced the VGG-16 pre-trained models using Keras (27) and fine-tuned this model following our dataset. The complete model architecture and hyperparameter details are shown in Table 3.

**Table 3:** Hyperparameter details used in the VGG-16 model according to our dataset.

**Layer (type)**	**Output shape**	**Param**
Vgg1 (Functional)	(None, 7, 7, 512)	14,714,688
flatten_ 1 (Flatten)	(None, 25,088)	0
dense_5 (Dense)	(None, 1,024)	25,691,136
dense_6 (Dense)	(None, 512)	524,800
dense_7 (Dense)	(None, 256)	131,328
dropout_ 1 (Dropout)	(None, 256)	0
dense_8 (Dense)	(None, 128)	32,896
dense_9 (Dense)	(None, 2)	516

Our VGG-16 model consists of a dense layer in which each neuron in the preceding layer sends signals to the neurons in the thick layer, which multiply matrices and vectors. A matrix-vector product's standard formula is shown in Equation 7.


(7) $$\begin{array}{l} A_{X}=\;\left[\begin{array}{ccc} a_{11} & a_{12} & a_{1n}\\ a_{21} & a_{22} & a_{2n}\\ a_{m1} & a_{m2} & a_{mn} \end{array}\right]\;[\begin{array}{c} x_{1}\\ x_{2}\\ x_{n} \end{array}]\;=\;\left[\begin{array}{ccc} a_{11}x_{1}+ & a_{12}x_{2} & a_{13}x_{3}\\ a_{21}x_{1} & a_{22}x_{2} & a_{23}x_{3}\\ a_{m1}x_{1} & a_{m2}x_{2} & a_{mn}x_{n} \end{array}\right] \end{array}$$

where *x* is a matrix with a diagonal of 1, and α is a (*M* × *N*) matrix. The values inside the matrix are the trained parameters of the earlier layers, and backpropagation can likewise be used to update them. We assessed our model's performance using accuracy, loss graphs, and ROC curves as described in Equations 8–10.


(8) $$\begin{array}{l} Accuracy=\;\frac{TP+TN}{TP+TN+FP+FN} \end{array}$$

where true positive (TP) is the total number of accurately identified positive cases; the number of accurately categorized negative cases (cases without stenosis) is known as true negative (TN); false positive (FP) and false negative (FN) are the numbers of false positive and false negative instances with ground truth, that is, respectively, classified as positive and negative (28).


(9) $$\begin{array}{l} L\left(W\right)=\;-\frac{1}{n}\;\overset{N}{\underset{n=1}{\sum }}\left(y_{n}\log\left(y_{n}\right)+\left(1-y_{n}\right)\log\left(1-y_{n}\right)\right) \end{array}$$

where *y* is the input patch's ground truth label, and calculating the gradient of the function *L* for the network weights *W* minimizes the loss function during the model training process. The receiver operating characteristics (ROC) curve (29), used to assess a test's overall diagnostic performance, compares the performance of two or more diagnostic tests. The area under the ROC, commonly referred to as ROC-AUC, can be interpreted as shown in Equation 10.


(10) $$\begin{array}{l} P\left(x_{1}>\;x_{0}\right)=P\left(x_{1}-\;x_{0}>0\right) \end{array}$$

where *x*_1_ and *x*_0_ are the continuous random variable giving the “score” output by our binary classifier for randomly chosen positive and negative samples. The ROC curve shows the trade-off between sensitivity (TPR) and specificity (FPR), represented in Equations 11, 12, respectively.


(11) $$\begin{array}{lll} TPR & = & \;\frac{TP}{p} \end{array}$$

(12) $$\begin{array}{lll} FPR & = & \;\frac{FP}{N} \end{array}$$

Pérez-Fernández et al. (30) by the definition of *TPR*, it corresponds to the probability of correctly classifying a randomly chosen positive sample, so *TPR (T)* = *P(X1* > *T)* = *1 – P(X1* ≤ *T)* = *1–F1(T)* by definition of the density function as shown in Equation 13.


(13) $$\begin{array}{l} TPR\left(T\right)=\;\int_{T}^{+\infty }f_{1}\left(x\right)dx \end{array}$$

By definition, ROC is shown in Equation 14.


(14) $$\begin{array}{l} ROC-AUC=\;\int_{0}^{1}TPR\;\left(FPR\right)\;dFPR\;\\ \;=\int_{0}^{1}TPR\;\left(FPR^{-1}\left(x\right)\right)\;dx \end{array}$$

By changing a variable, the integral equation becomes as shown in Equation 15.


(15) $$\begin{array}{l} \int_{+\infty }^{-\infty }TPR\;\left(T\right)\;X\;\left(-f_{0}\left(T\right)\right)\;dT=\;\int_{-\infty }^{+\infty }TPR\;\left(T\right)\;X\;f_{0}\;(T)dT \end{array}$$

By summarizing Equation 15, it becomes as shown in Equation 16.


(16) $$\begin{array}{l} \int_{-\infty }^{+\infty }\int_{T}^{+\infty }f_{1}\;\left(x\right)\;dx\;X\;f_{0}(T)\;dT \end{array}$$

By using this change in the variable for the inner integral, as shown in Equation 18, we obtained Equation 18.


(17) $$\begin{array}{lll} v & = & x-T \end{array}$$

(18) $$\begin{array}{l} \int_{-\infty }^{+\infty }\int_{0}^{+\infty }f_{1\;}\;\left(v+T\right)\;dv\;Xf_{0}\left(T\right)\;dT=\int_{0}^{+\infty }\int_{-\infty }^{+\infty }f_{1\;}\;\left(v+T\right)\;\\ \;X\;f_{0}\left(T\right)\;dT\;dv \end{array}$$

In general, the change in the variable for the inner integral is *u* = *v*+*T*, and the equation becomes as shown in Equation 19.


(19) $$\begin{array}{l} \int_{0}^{+\infty }\int_{-\infty }^{+\infty }f_{1\;}\;\left(u\right)\;X\;f_{0}\left(u-v\right)\;du\;dv \end{array}$$

According to the convolution theorem and assuming the convergence, with a density of *X1–X0* = *X1*+ *(–X0)*, our ROC-AUC equation becomes as shown in Equation 20.


(20) $$\begin{array}{l} P\;(x_{1}>\;x_{0})\;=\;P\;(x_{1}>\;x_{0})>0\;\\ \;=\;\int_{0}^{+\infty }\int_{-\infty }^{+\infty }f_{1}\left(u\right)\;X\;f_{0}\;(u-v)\;du\;dv \end{array}$$

## 3. Experimental results and discussion

A Doppler ultrasound is a non-invasive diagnostic that uses circulating red blood cells to reflect high-frequency sound waves (ultrasound) to assess the hemodynamic characteristics of blood flow *via* the cardiovascular vessels. The hemodynamic quantification of cardiovascular flow is vital to evaluating plaque rupture (30, 31), and medical imaging using ultrasound can obtain such information. By monitoring the rate of change in frequency, a color Doppler ultrasound may calculate the frequency of blood flow. A sonographer with ultrasound imaging training presses down on your skin with a small, handheld tool (transducer) while moving it over the area of your body being examined. Studies have shown that hypoechoic plaques are more prone to stroke than isoechoic and hyperechoic plaques (5). However, the detection of ulcer plaque and intraplaque hemorrhage by conventional ultrasound is limited, and the nature of plaque mainly depends on the operator's experience and subjective judgment, which has certain limitations. There is a need for a computer-aided autonomous framework to improve color Doppler efficiency and quality in daily clinical practice (31). Deep learning technology has recently permeated several areas of medical study and has taken a center stage in modern science and technology. In this research, we collected data from the Second Affiliation of Fujian Medical University, including stable cases and vulnerable cases.

**Figure 5:**
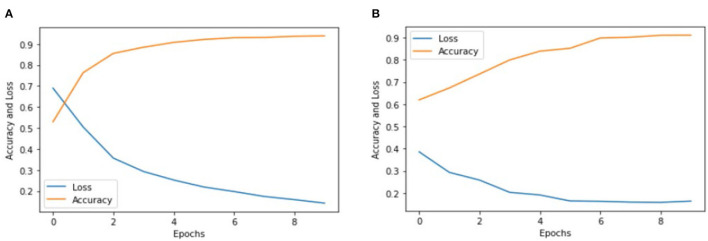
Accuracy and loss graph using the Inception V3 and VGG-16. **(A)** Representing the training accuracy and loss by using Inception V3 and **(B)** representing the training accuracy and loss of the VGG-16 models according to our dataset.

We implemented two pre-trained deep learning models, which include the Inception V3 and VGG-16, to classify color Doppler ultrasound images into two categories: one is high-risk carotid vulnerable plaque, and the second is stable carotid plaque. We fine-tuned (32) pre-trained deep learning models according to our dataset to classify color Doppler ultrasound images into two categories. Our trained Inception V3 model achieved 93.81% accuracy, and VGG-16 achieved 91.13% accuracy in classifying color Doppler ultrasound images into two categories. Figures 5A reports the training accuracy and loss by using Inception V3, and (Figure 5B) represents the training accuracy and loss by using VGG-16, respectively, according to our dataset. ROC curves are typically used to graphically represent the relationship or trade-off between clinical sensitivity and specificity for each potential cutoff for a test or combination of tests.

**Figure 6:**
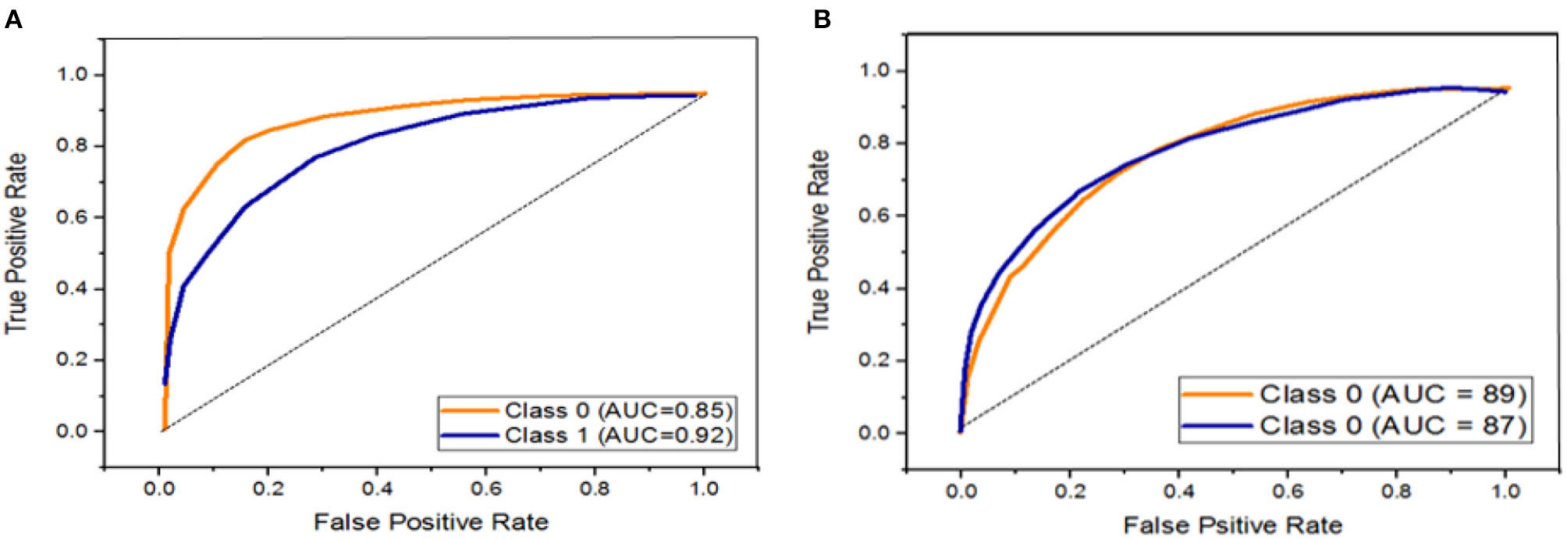
ROC curves for Inception V3 and VGG-16. **(A)** Representing the ROC by using Inception V3 and **(B)** representing the ROC by using VGG-16.

Figures 5A, B show the training accuracy and loss of Inception V3 and VGG-16 models for eight epochs. We can observe that the accuracy of Inception V3 is higher than the VGG-16, and the model loss is also decreased from 70 to 20%. We used the same number of epochs and hyperparameters for both models, and the results indicate that the Inception V3 model can better classify color Doppler ultrasound images into two categories. Figure 6 represents the performance of two models by using the ROC curve. Here, Figure 5A illustrates the implementation of the Inception V3 model for classifying color Doppler ultrasound images.

Similarly, Figure 5B illustrates the performance of the VGG-16 model for classifying color Doppler ultrasound images into two categories. The probability curve known as the ROC curve effectively separates the “signal” from the “noise” by plotting the true positive rate (TPR) against the false positive rate (FPR) at various threshold values (33). The capacity of a classifier to differentiate between classes is measured by the area under the curve (AUC), which is used as a summary of the ROC curve. When AUC is near 1, the classifier can correctly distinguish all the positive and the negative class points (34). In Figure 6A, the AUCs for class 0 and class 1 are 0.85 and 0.92, respectively. Hence, Inception V3 performed very well in classifying color Doppler ultrasound images. Similarly, in Figure 6B, the AUC for both classes is far from 1, and there is a high chance that the classifier will be able to distinguish the positive class values from the negative class values.

**Figure 7:**
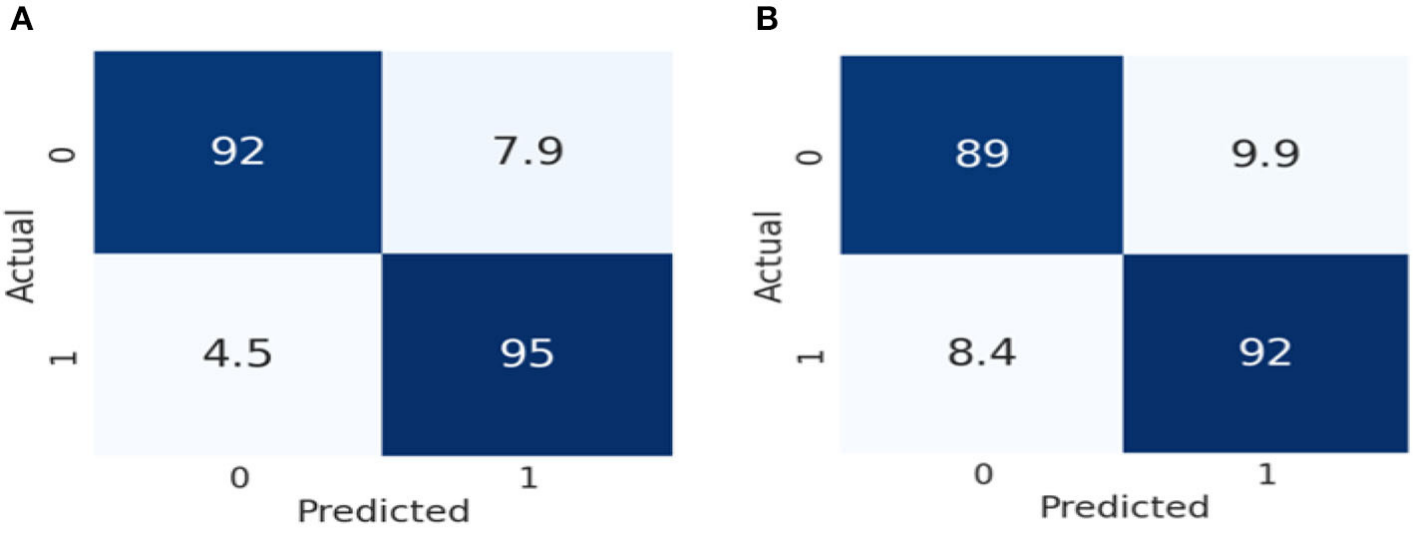
Confusion matrix representation of the performance of our models. **(A)** Represents the performance of Inception V3, while **(B)** illustrates the VGG-16 according to our dataset.

Figure 7 represents the performance of these two models by using the 2 × 2 confusion matrix. Here Figure 7A illustrates the performance of the Inception V3 model for classifying color Doppler ultrasound images. Similarly, Figure 7B represents the performance of VGG-16 in terms of the confusion matrix to classify color Doppler ultrasound images into high-risk, vulnerable, and stable carotid plaque.

As we can observe in Figure 7A, the Inception V3 model classifies 92% of the data into true positive (TP), meaning 92% of the data belongs to this class. 7.9% of the data was negative but falsely predicted as positive, 4.5% of data was positive but falsely predicted as negative, and 95% of the data was negative and also predicted as negative by the Inception V3 model. Similarly, in Figure 7B, the VGG-16 models classify 89% of the data into true positive (TP), meaning 89% of the data belongs to this class. 9.9% of the data was negative but falsely predicted as positive, 8.4% of the data was positive but falsely predicted as negative, and 92% was negative and also predicted as negative by the VGG-16 model. From the result, we can conclude that Inception V3 performs better than the VGG-16 in classifying color Doppler ultrasound images. In VGG-16, the fifth block is the first tuned block, followed by backward tuning until the first block represents the whole network (35). Whereas, the Inception V3 model has 11 inception model blocks, backward tuning starts from the mixed 10 inception module and then to the entire basic convolutional network (36). The Inception V3 model performs better in classifying color Doppler ultrasound images. We fine-tuned these two models according to our dataset to classify color Doppler images into two categories. Our trained Inception V3 achieved 93.81% accuracy VGG-16 model which gained 91.13% accuracy in classifying color Doppler images. Furthermore, we have compared our proposed framework performance with the previously proposed approach, as shown in Table 4.

**Table 4:** Comparative accuracy of the proposed approach with previous proposed studies.

**References**	**Approach**	**Accuracy %**
Yekkala et al. (37)	Ensemble machine learning	92
Wankhede et al. (38)	DL models	92.5
Uddin and Halder (39)	ML models	91.6
Our proposed framework	Pre-trained models	93.81

## Conclusion

In this study, we concluded that deep learning-based methods for carotid risk assessment are the most promising and successful. Our trained Inception V3 model achieved 93.81% accuracy, and the VGG-16 achieved 91.13% accuracy in classifying color Doppler ultrasound images. We assessed and compared our model's performance with previous methods using accuracy, loss graphs, and ROC curves, showing that our proposed framework outperformed other methods. Our framework will avoid inaccurate diagnoses caused by inadequate image quality and individual experience. Thus, the assessments in this study have shown that this methodology performs reasonable results for Doppler ultrasound image classification. In future implementations, extreme learning may be used as a more advanced classifier for plaque classification problems. Due to differences in patients and the appearance of the prostate, future studies should focus on testing the model with a larger dataset. Therefore, even though the results of studies have the potential for deep learning associated with different kinds of images, additional studies may need to be carried out clearly and transparently, with database accessibility and reproducibility, in order to develop useful tools that aid health professionals. In addition to the stenosis appearing in the carotid bifurcation and related arteries, the blockage of heart vessels may also occur, and this sometimes leads to left atrial enlargement [aa], and detailed analysis is required to understand the reason behind the blockage and to find effective ways to rectify the cardiac health problems (40).

## Data availability statement

The original contributions presented in the study are included in the article/supplementary material, further inquiries can be directed to the corresponding author/s.

## Ethics statement

The studies involving human participants were reviewed and approved by the Ethics No. of [2022] No. 288 of the Second Affiliated Hospital of Fujian Medical University. The patients/participants provided their written informed consent to participate in this study. Written informed consent was obtained from the individual(s) for the publication of any potentially identifiable images or data included in this article.

## Author contributions

S-SS proposed the study, discussed the contents with medical experts, and wrote the paper. L-YL performed the experiments, checked the equipment, and studied the results. YW checked all experimental image results and prepared the statistics in the paper. Y-ZL suggested improvements, modified parts of the experiment, and checked the entire manuscript. All authors contributed to the article and approved the submitted version.
